# Mutual Information-Based Brain Network Analysis in Post-stroke Patients With Different Levels of Depression

**DOI:** 10.3389/fnhum.2018.00285

**Published:** 2018-07-17

**Authors:** Changcheng Sun, Fei Yang, Chunfang Wang, Zhonghan Wang, Ying Zhang, Dong Ming, Jingang Du

**Affiliations:** ^1^Rehabilitation Medical Department, Tianjin Union Medical Centre, Tianjin, China; ^2^Department of Health and Exercise Science, Tianjin University of Sport, Tianjin, China; ^3^Rehabilitation Medical Department, Tianjin University of Traditional Chinese Medicine, Tianjin, China; ^4^Department of Biomedical Engineering, College of Precision Instrument and Opto-Electronics Engineering, Tianjin University, Tianjin, China

**Keywords:** post-stroke depression (PSD), electroencephalography (EEG), mutual information (MI), graph theory, brain network

## Abstract

Post-stroke depression (PSD) is the most common stroke-related emotional disorder, and it severely affects the recovery process. However, more than half cases are not correctly diagnosed. This study was designed to develop a new method to assess PSD using EEG signal to analyze the specificity of PSD patients' brain network. We have 107 subjects attended in this study (72 stabilized stroke survivors and 35 non-depressed healthy subjects). A Hamilton Depression Rating Scale (HDRS) score was determined for all subjects before EEG data collection. According to HDRS score, the 72 patients were divided into 3 groups: post-stroke non-depression (PSND), post-stroke mild depression (PSMD) and post-stroke depression (PSD). Mutual information (MI)-based graph theory was used to analyze brain network connectivity. Statistical analysis of brain network characteristics was made with a threshold of 10–30% of the strongest MIs. The results showed significant weakened interhemispheric connections and lower clustering coefficient in post-stroke depressed patients compared to those in healthy controls. Stroke patients showed a decreasing trend in the connection between the parietal-occipital and the frontal area as the severity of the depression increased. PSD subjects showed abnormal brain network connectivity and network features based on EEG, suggesting that MI-based brain network may have the potential to assess the severity of depression post stroke.

## Introduction

Post-stroke depression (PSD) is among the most frequent neuropsychiatric consequences of cerebral ischemia (Cojocaru et al., 2013). PSD is an abnormal negative emotional response caused by loss, disappointment or failure. PSD has a significant negative impact on the rehabilitation of stroke (Ghose et al., 2005), thus seriously affecting the patient's future quality of life (Bays, 2001; Ayerbe et al., 2013; Chen et al., 2014) and delaying or even hindering the process for rehabilitation and return to society. Approximately one-third of stroke patients have aphasia (Berthier, 2005; Engelter et al., 2006), and approximately 70% will have cognitive impairment (Nys et al., 2007). Aphasia and cognitive impairment make it difficult to obtain the changes in patients' emotions and interests, which pose a great challenge for the diagnosis of PSD. There are few guidelines for the assessment, treatment and prevention of PSD (Babkair, 2017), and more than half cases are not correctly diagnosed.

Depression was thought to be the result of a dysregulation in the ability of brain cells to communicate with each other (Cai et al., 2013). Researchers have found abnormalities in the transmission of excitatory signals between cells in depression. Restoring normal brain communication is one mechanism underlying the successful function of antidepressant drugs such as serotonin, which is a key factor in depression remission (Cai et al., 2013). Disrupted network connectivity has been found in some core major depressive disorder (MDD) networks (Brakowski et al., 2017). Previous findings in geriatric depression have also strongly suggested “brain network dysfunction” as the best explanatory model for understanding the biological mechanism of depression (Drevets et al., 2008). All of the possible etiologies of late-life depression result in different depressive symptoms by disturbing the dynamics and functions of different brain networks (Tadayonnejad and Ajilore, 2014). Impairment of the affective regulatory pathway has been suggested as a possible pathogenic factor related to vascular disease according to previous studies (Alexopoulos et al., 1997a,b). We suggest that PSD patients' abnormal connectivity among brain areas could be driving this pathogenesis, which may appear as “disconnection” symptoms.

Functional connectivity in the human brain can be represented as a network using electroencephalography (EEG) signals (Rathee et al., 2017). One of the functional connectivity measures for analyzing EEG is Mutual information (MI) which is a non-directional connectivity measure. It enables the estimation of both linear and non-linear statistical dependencies between time series and can be used to detect functional coupling (Wang et al., 2009). Because neural dynamics almost certainly includes many highly nonlinear processes, MI analysis may be helpful in understanding and quantifying the nonlinear transmission of information within the brain (Jeong et al., 2001). Abnormal cortical connections using MI have been found in nervous system diseases, such as Alzheimer's, schizophrenia and Parkinson's (Coronel et al., 2017; Yin et al., 2017).

Graph theory has played an integral role in recent efforts to understand the function of complex systems including brain networks. Importantly, graph-based representations of brain networks can quantitatively describe the connectivity of different brain regions. It has been applied to understand brain networks and emerged as a powerful analytic tool for brain connectivity. Using this method, many researchers have studied the structural and functional networks of the brain and the network anomalies caused by neuropsychiatric disorders (Schreiber, 2000; Bernhardt et al., 2013; Rathee et al., 2017). In brain networks, different connections represent different paths of information transfer. This study aimed to analyze the features of MI-based undirected and weighted brain network to explore the abnormal brain connectivity of the stroke patients with different degrees of depression.

## Materials and methods

### Participants

This study was performed in the Department of Rehabilitation, Tianjin Union Medical Center, Tianjin, China. All participants were right-handed and native speakers of Mandarin Chinese. The hospital ethics committee approved the study. All participants were informed of the aims and protocols of the experiments.

This study involved 35 healthy controls (HC) and 72 stroke patients. The HC group had no history of neurological or psychiatric disease. All patients were divided into three groups based on their Hamilton Depression Rating Scale (HDRS) score. The patients in the post-stroke non-depression group (PSND), post-stroke mild-depression group (PSMD), and post-stroke depression group (PSD) have HDRS scores of ≤ 5, 6–20, and >20, respectively. Other demographic and general subject characteristics are listed in Table 1.

**Table 1:** Demographic and clinical features of four groups.

**Variables**	**Healthy controls (HC, *n* = 35)**	**Stroke patients (*****n*** = **72)**			***F***	***df***	***p***
		**PSND (HDRS ≤ 5, *n* = 14)**	**PSMD (5 < HDRS ≤ 20, *n* = 43)**	**PSD (HDRS > 20, *n* = 15)**			
Age [M ± SD (years)]	50.25 ± 15.01	59.36 ± 8.93	60.39 ± 8.85	62.73 ± 6.27	2.074	3/103	0.108
Sex (male/female)	19/16	11/3	27/16	8/7	1.014	2/69	0.368
Handedness (left/right)	3/32	1/13	3/40	0/15	0.425	3/103	0.736
HDRS [M ± SD (score)]	2.23 ± 1.14	3.36 ± 1.78	11.77 ± 4.51	28.40 ± 7.90	150.44	3/103	0.000^*^
Time after stroke [M ± SD (months)]		2.82 ± 2.58	3.19 ± 5.96	5.44 ± 8.26	0.913	2/69	0.406
Lesion location (Left/Right)		7/7	24/19	6/9	0.548	2/69	0.580

* *p < 0.05*.

### EEG recording and preprocessing

The subjects were seated in a resting state with their eyes closed for 5 min in a quiet environment. The EEG was recorded at 16 scalp loci (Fp1, Fp2, F3, F4, F7, F8, C3, C4, T3, T4, P3, P4, O1, O2, T5, and T6) in compliance with the international 10–20 system using a NicoletOne digital video electroencephalograph made by US. The skin resistance at each site was <10 kΩ. EEG data were collected for 300 s at a rate of 250 Hz. Data containing artifacts were removed in an off line analysis. We also used independent component analysis (ICA) to identify and remove residual ocular activity (Fanciullacci et al., 2017). The EEG signals were re-referenced to the bilateral mastoid electrodes (A1 and A2), and removed each channels baseline from continuous EEG data by using the routine pop_rmbase (EEGLAB). Then a Hamming windowed sinc FIR filter was used to filter the data with a bandwidth of 0.1–100 Hz by using the routine pop_eegfiltnew (EEGLAB).

As previous studies have proved that the infinity reference was proper for EEG network analysis (Qin et al., 2010), we changed linked earlobes to infinity reference using a reference electrode standardization technique (REST) (Dong et al., 2017; Yao, 2017). REST is used for the approximate standardization of the reference of scalp EEG recordings to a point at infinity that, being far from all possible neural sources, acts like a neutral virtual reference(Marzetti et al., 2007). Numerous studies have shown that REST is the most accurate reference method for brain network analysis (Yao, 2001; Qin et al., 2010). A REST toolbox which developed by Dong et al. (2017) were used in this study.

### Multivariate causal analysis of data

In information theory, MI is a measure of the statistical dependence between two random variables (Ince et al., 2017). The average amount of information obtained from any observation of X = {*x*_*i*_} is the entropy H of a system:

$$\begin{array}{l} H\left(\text{X}\right)=-\underset{x_{i}}{\sum }P_{X}\left(x_{i}\right)\log P_{X}\left(x_{i}\right) \end{array}$$

where *P*_*X*_ (*x*_*i*_) is the probability that an isolated measurement will find the system in the *i*th element of the bin. We evaluated these probabilities *P*_*X*_(*x*_*i*_) by constructing a histogram (from 1,250 data points) of the variations of the measurement *x*_*i*_.

Before any measurement of X, this information is called uncertainty. Under the condition *Y* = *y*_*j*_, *H*(*X*) has to be replaced by the conditional uncertainty on X

$$\begin{array}{l} H\left(\text{X}|\text{Y}=y_{j}\right)=-\underset{x_{i}}{\sum }\frac{P_{XY}\left(x_{i},y_{j}\right)}{P_{Y}\left(y_{j}\right)}\log\frac{P_{XY}\left(x_{i},y_{j}\right)}{P_{Y}\left(y_{j}\right)} \end{array}$$

Where *P*_*XY*_(*x*_*i*_, *y*_*j*_) is the joint probability density for the measurements of X and Y that produce the values X and Y. *H*(*X*|*Y* = *y*_*j*_) indicates the amount of uncertainty in a measurement of *x*, given that y has been measured and found to be *y*_*j*_. From this, we get the mean conditional uncertainty on X over *y*_*j*_, under the condition that Y is known

$$\begin{array}{l} H\left(\text{X}|\text{Y}\right)=-\underset{x_{i},y_{j}}{\sum }P_{XY}\left(x_{i},y_{j}\right)\log\left[P_{XY}\left(x_{i},y_{j}\right)/P_{Y}\left(y_{j}\right)\right]\\ =H\left(\text{X},\text{Y}\right)-H\left(\text{Y}\right) \end{array}$$

where

$$\begin{array}{l} H\left(\text{X},\text{Y}\right)=-\underset{x_{i},y_{j}}{\sum }P_{XY}\left(x_{i},y_{j}\right)\log\left[P_{XY}\left(x_{i},y_{j}\right)\right] \end{array}$$

So we define the MI as the amount by which a measurement of *Y* reduces the uncertainty of *X*. The MI is as follows: *MI*_*XY*_ = *H*(*X*)−*H*(*X*|*Y*) = *H*(*X*)+*H*(*Y*)−*H*(*X, Y*) = *MI*_*YX*_which can be rewritten as:

$$\begin{array}{l} {MI}_{XY}=MI_{YX}==-\underset{x_{i},y_{j}}{\sum }P_{XY}\left(x_{i},y_{j}\right)\log\frac{P_{XY}\left(x_{i},y_{j}\right)}{P_{X}\left(x_{i}\right)P_{Y}\left(y_{j}\right)} \end{array}$$

MI has the maximum value when the two time series are completely the same. If one system is completely independent of the other, the MI is zero (Na et al., 2002). The principal difficulty in calculating the MI from experimental data is estimating *P*_*XY*_(*x, y*) from histograms, selecting different sampling bins has a great influence on the accuracy of MI (Jeong et al., 2001). In this study, we took logarithm with base e, and 11 bins were adopted for 1,250 samples, which can provide a stable estimate.

In this study, EEG data were segmented into 5-s epochs (1,250 data points), and a total of 60 epochs for each channel were analyzed using the MI. 60 MI values can be obtained between any two channels, and the mean index value of the 60 MIs were performed as the final MI indices. All routines above were implemented in MATLAB (MathWorks, Inc.).

### Graphical description of the network

Graph theory has proven very useful in statistics as a way to describe the dependent relations between random variables (Salvador et al., 2005). In graph theory, a network is reduced to an abstract description as a set of nodes connected by edges (or lines) (Bassett and Bullmore, 2009). The edges can be directed or undirected and weighted or unweighted.

**Figure 1:**
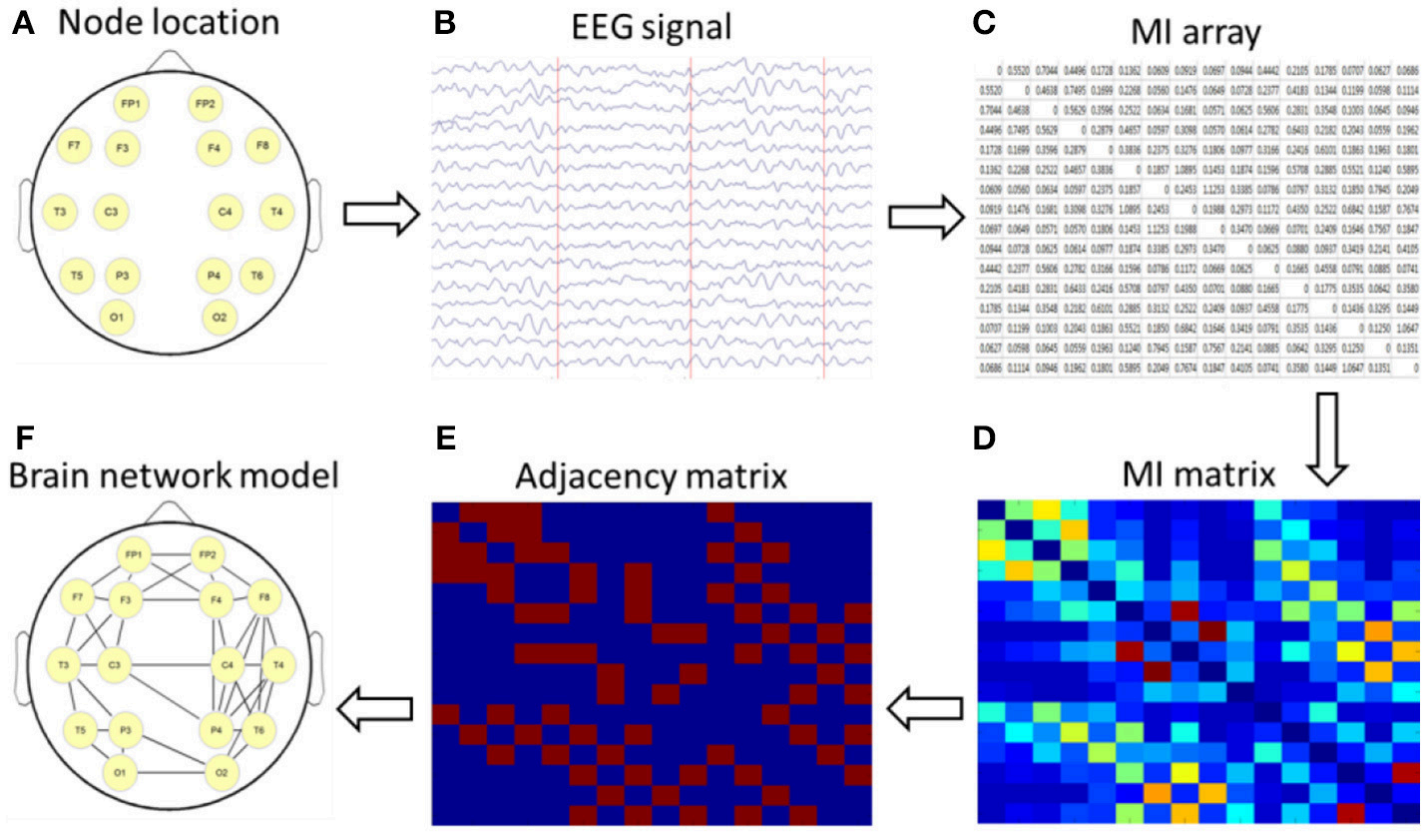
Schematic diagram of a brain network based on mutual information (MI) technology. **(A)** nodes location; **(B)** acquisition of 16-lead EEG data; **(C)** calculation of MI; **(D)** formation of a MI matrix; **(E)** adjacency matrix with a certain threshold; **(F)** the brain network diagram was formed according to the relationship among the 16 leads.

The nodes and edges of a brain graph can be empirically defined in many ways. In this study, we used 16 leads as nodes and constructed the cortical undirected network graph by using the calculated MI as the edge of the network. By using the topological properties of networks, we analyzed the characteristics of brain networks in different subjects and then explored the abnormal connectivity of the brain in patients with depression after stroke. The setup process for the brain network is shown in Figure 1.

Topological properties of a brain network can be described using some graph measures based on Graph theory, such as clustering coefficient and betweenness centrality. Clustering coefficient is one key topological metric which quantifies degree of collectivization of one network. The clustering coefficient of one node measures the connecting size of its adjacent edges. The calculation formula for clustering coefficient *C*_*i*_ of node *i* is shown

(1) $$\begin{array}{l} C_{i}=\frac{e_{i}}{C_{k}^{2}}=\frac{2e_{i}}{k_{i}\left(k_{i}-1\right)} \end{array}$$

Where, *k*_*i*_ is the number of all adjacent nodes of node i, *e*_*i*_ is the number of connected edges between all neighboring nodes of node i. One node had value 0, while which only has a neighbor or none. The mean clustering coefficients of all the nodes represent the network's coefficient. The betweenness centrality is used to describe the role and status of one node to the network. Higher betweenness centrality indicates more important status and the corresponding node is a core node for the network. The caculation formula for betweeness centrality is shown

(2) $$\begin{array}{l} N_{i}=\underset{j\;\neq i\;\neq k\;\epsilon \;G}{\sum }\frac{\sigma_{jk}\left(i\right)}{\sigma_{jk}} \end{array}$$

σ_*jk*_(*i*) is the number of shortest path from node j to node k, which passing node i. In this study, clustering coefficients and betweenness centrality were calculated by binary MI matrices (elements above the threshold were defined as 1, otherwise defined as 0) at each threshold.

### Simulation of MI-based brain network

**Figure 2:**
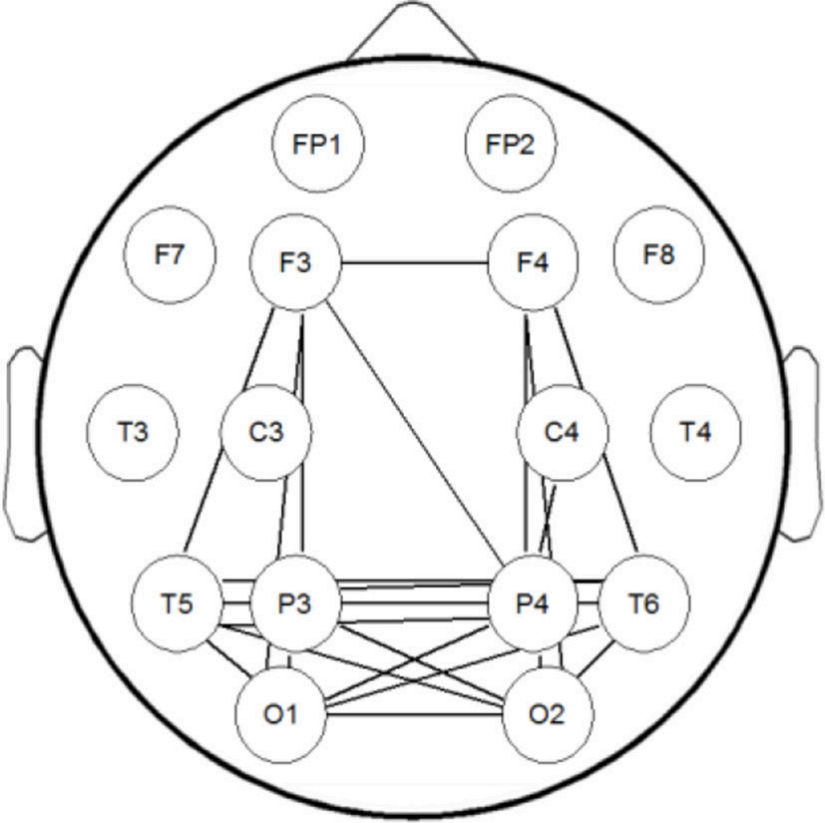
The brain network of the simulation dataset. Information flow from the left (below C3) to the right (below C4) source is modeled by means of a bivariate AR model.

Using MI to assess statistical dependence between two EEG signals, there can be contamination of spurious connectivity caused by volume conduction. In order to solve this problem, we used a surrogate data method to conduct a simulation study. We generated a dataset which has the same structure with our EEG data using Matlab code provided by Stefan Haufe et al. (Fonov et al., 2009, 2011; Haufe et al., 2013). In this dataset a linear time-lagged information flow from the left hemisphere (brain area below C3) to the right hemisphere (area below C4) is simulated by means of a bivariate AR model. This flow is to be detected as the only true time-lagged interaction happening in the data. We have established the MI brain network of this dataset, the result is shown in Figure 2. It can be seen from Figure 2 that MI can better reflect the true connection between the corresponding brain regions and suppress spurious connectivity. This is basically consistent with the connectivity between simulated EEG sensor measurements estimated by phase-slope index (PSI) in Stefan Haufe et al. (2013). The difference between the two methods is that MI has no directionality, and PSI can reflect the direction of information flow.

## Results

As shown in Table 1, four groups showed no significant difference in other demographic and clinical features except for HDRS.

There are 16 channels' time series of 300 s duration for each subject. These time series were analyzed in sequential windows of 5 s duration, yielding 16 time series with a length of 60 epochs. For each subject, this approach yielded 120 unique MIs (from the 16 × 16 MI matrix removing diagonal and symmetric data).

**Figure 3:**
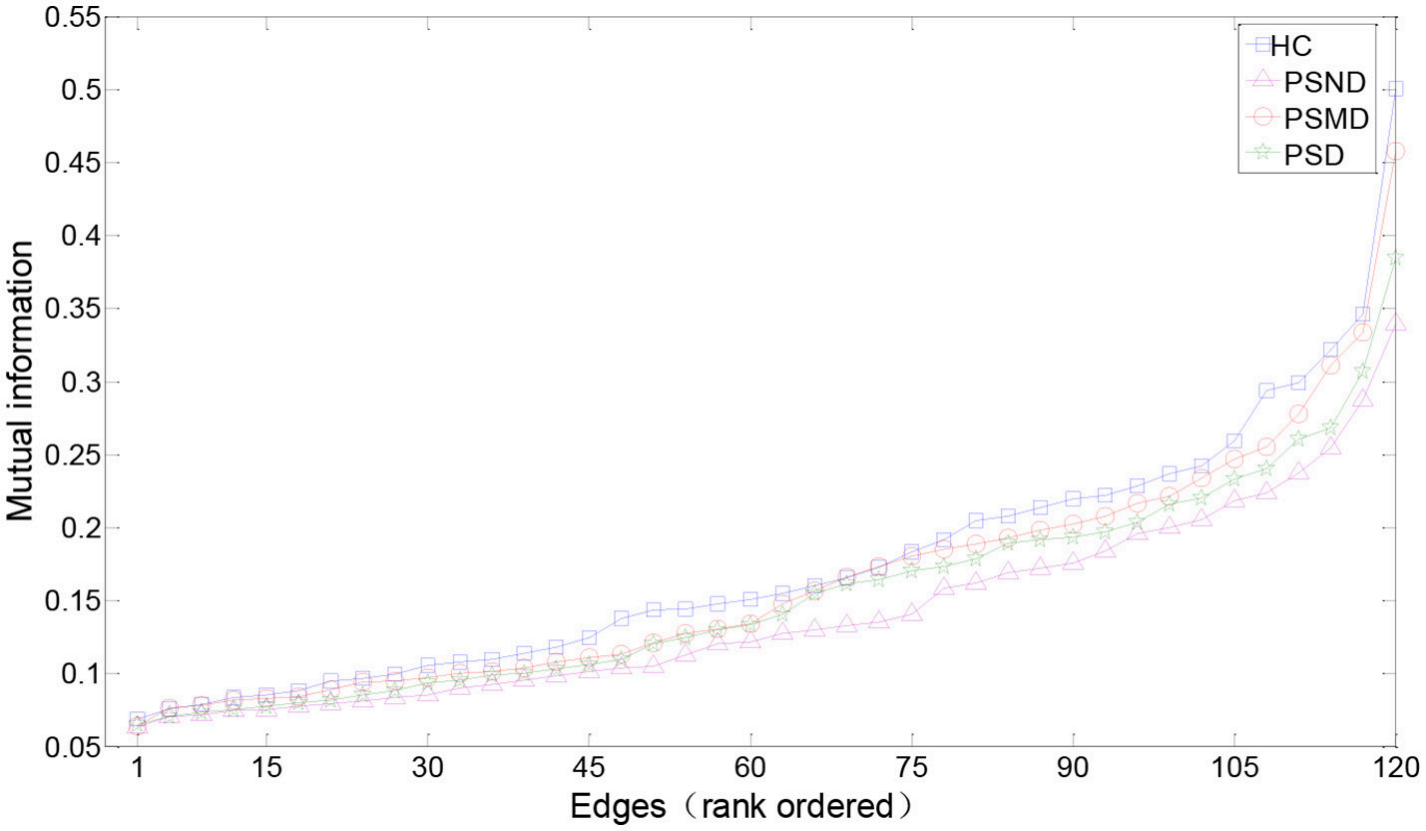
Edges from mean unthresholded MI matrices of four subject groups rank-ordered by MI values. There are a total of 120 unique correlations in each unthresholded MI matrix. The strongest 10–30% of these MIs are considered for subsequent graph analysis. MIs in healthy people are on average slightly greater than in the other three groups for a range of rank-ordered means.

Figure 3 shows the rank-ordered average MIs for unthresholded MI matrices of four groups. We can find that more than 70% of MIs are between 0.05 and 0.2, they capture only a small amount of the common variance (the square of the MI < 4%) in the underlying dynamics, and also the difference between the four curves in this range is not significant in the Figure 3. According to the study of Rubinov et al. (2009), the 10–30% of the strongest MIs are more likely to reflect the underlying network architecture. Selected a certain range is also more convenient for us to find patterns in complex brain networks, so the following analysis mainly focused on 10–30% of the strongest MIs. There is no significant difference between four groups in the Figure 3. MIs in healthy people are on average slightly greater than in the other three groups for a range of rank-ordered means.

**Figure 4:**
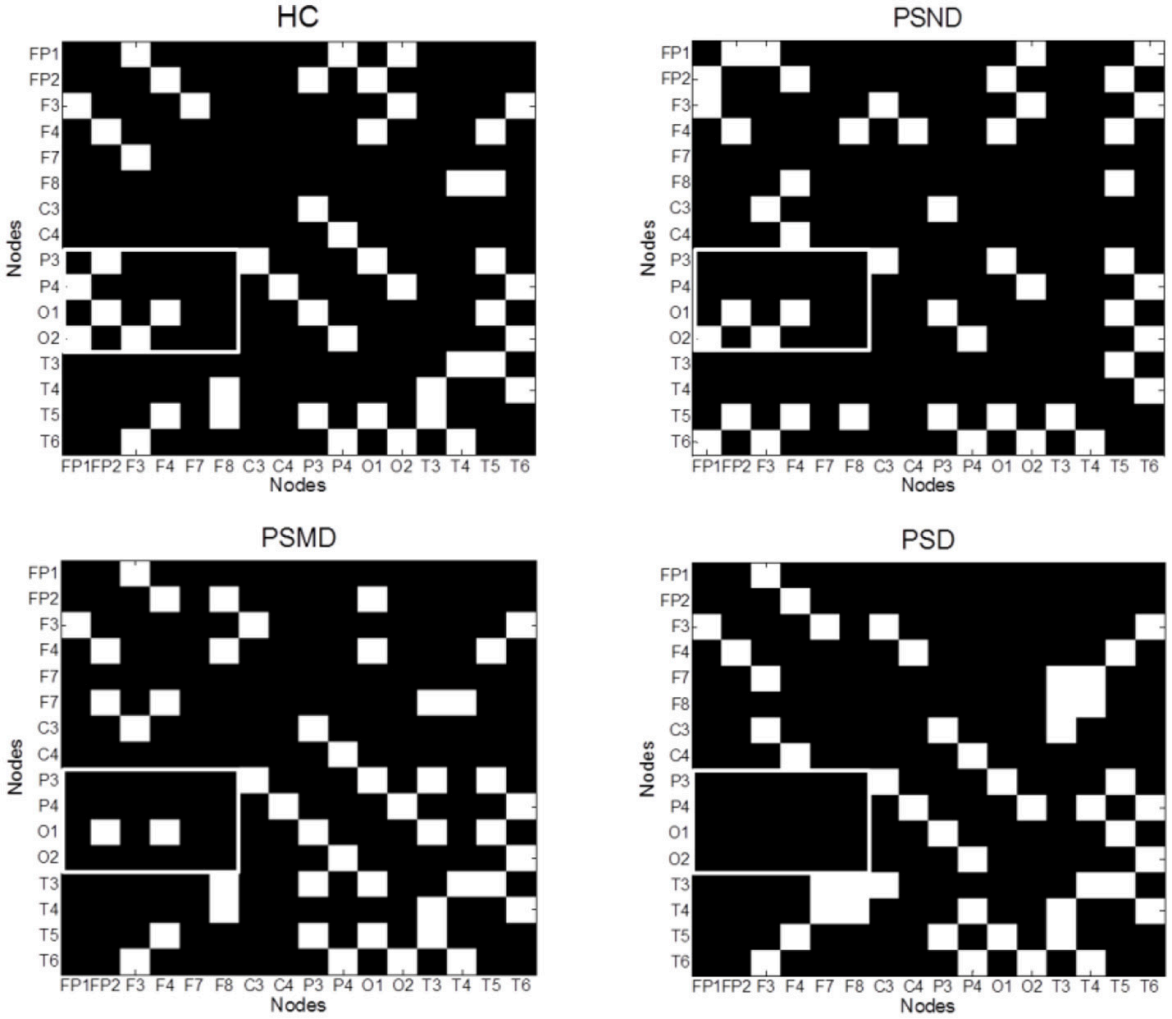
The average MI matrices of four subject groups thresholded such that only 20% of the strongest weights are preserved. The white matrix elements represent functional connectivity. The key difference areas are marked with boxes.

Figure 4 shows the average MI matrices for four groups, which were thresholded such that 20% of the strongest edges are presented. The MI matrices had the same number of elements after thresholding. The white matrix elements represent functional connectivity. The connection between the parietal-occipital area and the frontal area shows a decreasing trend as the severity of the disease increases (white squares in the Figure 4).

**Figure 5:**
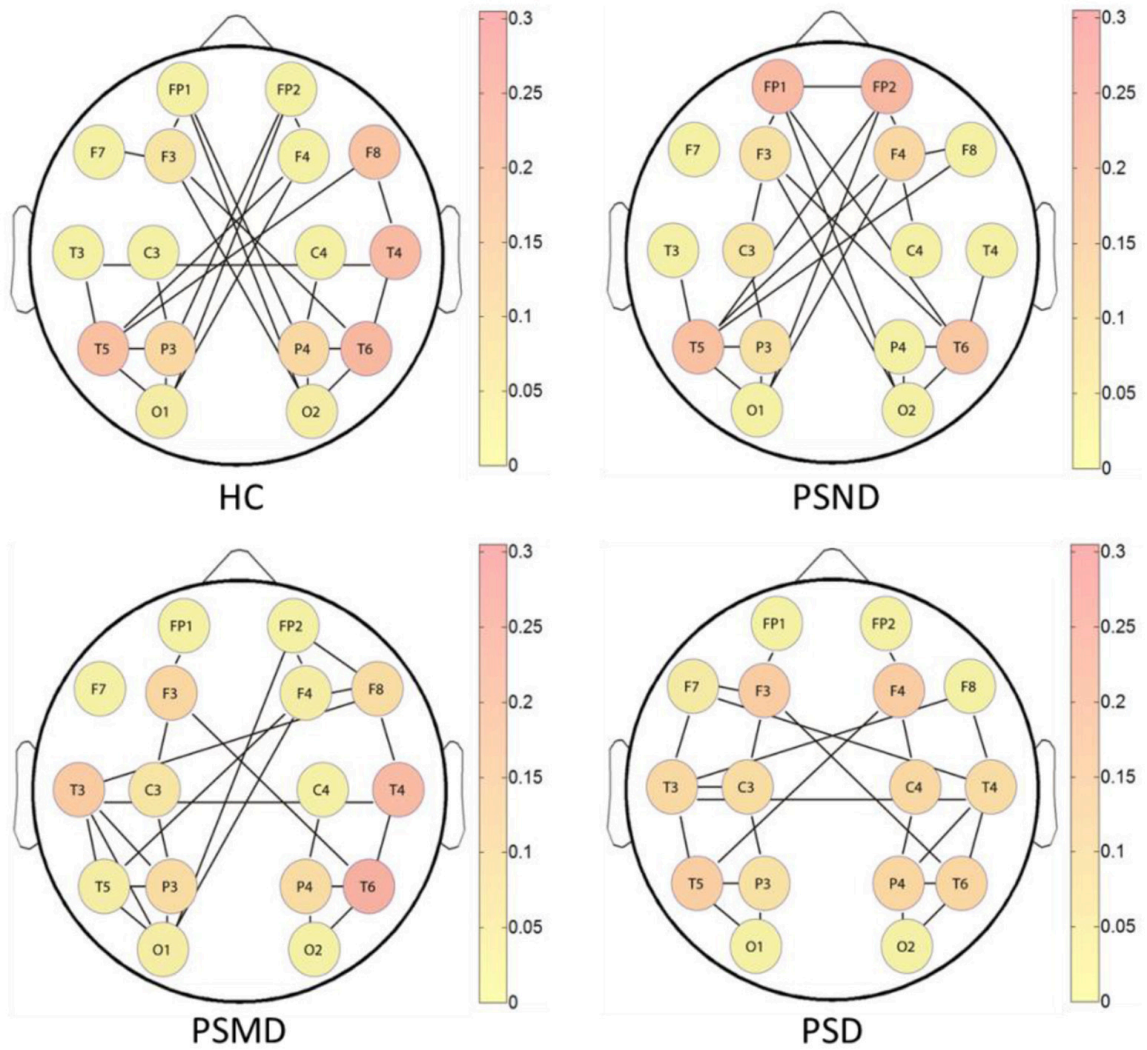
The brain networks based on average MI matrices of four subject groups at threshold 20% of the strongest edges. Different colors indicate the betweenness centrality of the each node.

Figure 5 shows the brain networks based on the average MI matrices of four groups thresholded such that only 20% of the strongest edges are preserved. Different colors represent the size of the betweenness centrality of the nodes, that is, the importance of each node in the network. The connection between the left and right brain is weakened as the degree of depression increases. And the internal connections of each hemisphere have been enhanced correspondingly.

The nodes color shows that the core nodes of PSD were more scattered than the other three groups. This may affect the degree of clustering between nodes, and the subsequent analysis proves this by the statistical results of clustering coefficients. The subsequent statistical analysis results showed that there was no significant difference in the betweenness centrality of each node among the four groups at almost all thresholds.

According to the brain network characteristics of Figure 5, we performed statistical analysis of the relevant topological properties. Post-stroke depressed subjects showed weaker connections between the left and right hemisphere. Table 2 shows the statistical significance of the edge numbers between left and right cerebral hemispheres across 10–30% thresholds, assessed at each threshold by One-Way ANOVA. *Post-hoc* group comparisons were performed using Least Significant Difference (LSD) or Tamhane's T2 (IBM SPSS Statistics 19), according to whether the variance meets the condition of homogeneity. The significant results (*p* < 0.05) are in bold. If the p values are all above 0.05 at all thresholds between two groups will not be shown here. As can be seen from the table, there are some significant differences between the HC group and the PSMD and the PSD group at about half number of the thresholds. In particular, there is a clear difference between HC and PSD at a large continuous threshold range (19–29%). We noticed no significant difference between the HC group and the PSND group, but there are differences between PSND and PSD at several thresholds.

**Table 2:** Edge numbers between left and right cerebral hemispheres at thresholds 10–30%.

**Threshold**	**Edges number (M** ± **SD)**				***p*****-value**		
	**HC**	**PSND**	**PSMD**	**PSD**	**HC vs. PSMD**	**HC vs. PSD**	**PSND vs. PSD**
0.10	3.57 ± 1.50	3.93 ± 1.98	3.07 ± 2.29	3.24 ± 1.73	0.371	0.426	0.206
0.11	4.29 ± 1.56	4.43 ± 2.16	3.47 ± 2.50	3.83 ± 1.88	0.180	0.317	0.191
0.12	5.69 ± 1.83	5.93 ± 2.28	4.33 ± 2.60	5.02 ± 2.03	**0.043**	0.178	**0.047**
0.13	6.26 ± 1.92	6.43 ± 2.38	4.87 ± 2.53	5.55 ± 2.05	**0.040**	0.156	0.056
0.14	6.83 ± 1.90	7.07 ± 2.31	5.40 ± 2.50	5.98 ± 2.17	**0.038**	0.093	**0.043**
0.15	7.57 ± 1.89	7.57 ± 2.26	6.07 ± 2.57	6.60 ± 2.22	**0.030**	0.057	0.071
0.16	8.11 ± 1.98	8.14 ± 2.13	6.73 ± 2.38	7.07 ± 2.20	**0.043**	**0.040**	0.086
0.17	9.34 ± 1.94	9.36 ± 2.41	8.00 ± 2.71	8.43 ± 2.24	0.060	0.084	0.113
0.18	9.89 ± 2.00	10.00 ± 2.36	8.53 ± 2.80	8.93 ± 2.14	0.057	0.069	0.086
0.19	10.60 ± 1.95	10.57 ± 2.23	9.20 ± 2.95	9.43 ± 2.35	0.057	**0.032**	0.120
0.20	11.20 ± 2.01	11.14 ± 2.36	9.73 ± 2.77	10.07 ± 2.39	**0.048**	**0.040**	0.113
0.21	11.83 ± 2.09	11.71 ± 2.34	10.60 ± 2.68	10.60 ± 2.26	0.092	**0.023**	0.203
0.22	13.17 ± 2.08	13.00 ± 2.36	11.80 ± 2.56	11.88 ± 2.27	0.057	**0.016**	0.165
0.23	13.80 ± 2.09	13.43 ± 2.29	12.73 ± 2.67	12.52 ± 2.27	0.140	**0.018**	0.422
0.24	14.57 ± 2.00	14.07 ± 2.15	13.33 ± 2.44	13.21 ± 2.28	0.077	**0.010**	0.378
0.25	15.14 ± 1.88	14.64 ± 2.35	13.80 ± 2.37	13.90 ± 2.32	0.055	**0.017**	0.314
0.26	15.74 ± 1.89	15.29 ± 2.49	14.27 ± 2.35	14.43 ± 2.24	**0.034**	**0.011**	0.221
0.27	17.00 ± 1.67	16.71 ± 2.60	15.47 ± 2.28	15.69 ± 2.13	**0.021**	**0.008**	0.118
0.28	17.63 ± 1.73	17.43 ± 2.67	16.07 ± 2.41	16.52 ± 2.13	**0.022**	**0.028**	0.095
0.29	18.23 ± 1.87	18.07 ± 2.69	16.93 ± 2.38	17.02 ± 2.05	0.057	**0.018**	0.163
0.30	18.66 ± 1.87	18.57 ± 2.97	17.67 ± 2.47	17.64 ± 2.15	0.162	0.055	0.288

*Significant results are in bold*.

Table 3 shows clustering coefficients in four groups with thresholds of 10–30% of strongest edges. The difference in clustering is significant at 11 of the 21 thresholds between HC and PSD, most assemble at higher thresholds, and there is no significant difference between any other two groups. The clustering of HC is higher than that of PSD in Table 3. It indicates that healthy people's EEG signals have a higher degree of clustering.

**Table 3:** Clustering coefficients at thresholds 10–30%.

**Threshold**	**Clustering coefficient (M** ± **SD)**				***p-*value**
	**HC**	**PSND**	**PSMD**	**PSD**	**HC vs. PSD**
0.10	0.25 ± 0.12	0.18 ± 0.11	0.26 ± 0.11	0.20 ± 0.11	0.080
0.11	0.27 ± 0.12	0.22 ± 0.11	0.26 ± 0.13	0.23 ± 0.13	0.200
0.12	0.31 ± 0.11	0.25 ± 0.11	0.27 ± 0.13	0.26 ± 0.12	0.062
0.13	0.34 ± 0.11	0.28 ± 0.12	0.29 ± 0.13	0.29 ± 0.13	0.050
0.14	0.35 ± 0.10	0.31 ± 0.09	0.32 ± 0.12	0.31 ± 0.12	0.124
0.15	0.36 ± 0.09	0.33 ± 0.10	0.33 ± 0.10	0.33 ± 0.13	0.237
0.16	0.38 ± 0.09	0.35 ± 0.09	0.34 ± 0.10	0.33 ± 0.12	**0.028**
0.17	0.41 ± 0.10	0.37 ± 0.08	0.37 ± 0.11	0.37 ± 0.11	**0.047**
0.18	0.42 ± 0.10	0.40 ± 0.08	0.38 ± 0.11	0.38 ± 0.10	0.067
0.19	0.43 ± 0.09	0.41 ± 0.08	0.42 ± 0.14	0.39 ± 0.10	0.071
0.20	0.46 ± 0.10	0.43 ± 0.08	0.44 ± 0.13	0.40 ± 0.10	**0.021**
0.21	0.48 ± 0.11	0.44 ± 0.09	0.45 ± 0.12	0.42 ± 0.10	**0.036**
0.22	0.50 ± 0.11	0.47 ± 0.08	0.46 ± 0.11	0.47 ± 0.11	0.155
0.23	0.53 ± 0.11	0.47 ± 0.09	0.48 ± 0.11	0.48 ± 0.11	**0.036**
0.24	0.53 ± 0.10	0.49 ± 0.09	0.51 ± 0.12	0.49 ± 0.10	**0.047**
0.25	0.54 ± 0.10	0.50 ± 0.09	0.52 ± 0.10	0.50 ± 0.10	**0.045**
0.26	0.55 ± 0.09	0.52 ± 0.09	0.52 ± 0.10	0.51 ± 0.10	**0.039**
0.27	0.56 ± 0.09	0.56 ± 0.09	0.53 ± 0.12	0.52 ± 0.08	**0.038**
0.28	0.57 ± 0.08	0.57 ± 0.10	0.54 ± 0.10	0.53 ± 0.08	0.104
0.29	0.58 ± 0.08	0.58 ± 0.10	0.56 ± 0.10	0.54 ± 0.08	**0.027**
0.30	0.59 ± 0.08	0.58 ± 0.09	0.56 ± 0.09	0.54 ± 0.08	**0.026**

*Significant results are in bold*.

## Discussion

In this study, we examined the brain network performance in post-stroke depressed patients using the EEG-MI. We found that stroke patients with different degrees of depression showed different connection features. These features may be helpful in the diagnosis of PSD. Our results showed significant weakened connections between the left and right cerebral hemispheres in stroke patients compared to those in healthy controls, and this feature is more obvious with the deepening of the degree of depression. This suggests that depression affects the information communication between the left and right hemispheres in stroke patients. Among the stroke patients, the core nodes of PSD were more scattered than the other three groups. The connections between the parietal-occipital area and the frontal area showed a decreasing trend as the severity of the depression increases. Post-stroke depressed patients have a lower clustering coefficient than healthy subjects, with a significant difference at one-half thresholds.

The basal ganglia proved to play key roles in cortical and subcortical connected circuits, including the frontal, premotor and motor networks (Draganski et al., 2008; Thomas, 2009; Lao et al., 2016). This area may receive multiple cortical inputs in the presence of oscillatory activity and produce a high frequency drive back to the cerebral cortex, especially the supplementary motor area (Williams et al., 2002). Dysfunction of the frontal-parietal-occipital network in stroke patients may result from an organic lesion of the basal ganglia.

For depressed patients following stroke, the interhemispheric interaction was found to be highly disturbed in this study. Yamada et al. (1995) found that depressed patients showed lower frontal interhemispheric coherences than normal controls in each EEG band, and EEG power and coherence in presenile and senile depression. Wei et al. (2010) get similar findings with the above research. Furthermore, they found the inter-hemispheric coherence was correlated with some emotional processing. A decreased interhemispheric modulation was found in patients with major depression (Bajwa et al., 2008; Wu, 2014), which is consistent with our findings. Slow interhemispheric switching mechanisms in mood disorders may explain the weakened hemispheric information flow in PSD patients.

The frontal lobe plays a regulatory role in emotional cognition, the connection between the parietal-occipital and the frontal was decreased in depression in this study. Previous studies have reported aberrant EEG performance, such as increased slow activity in the frontal areas (Grin-Yatsenko et al., 2009, 2010), in depressed patients. Depressed older adults were found to have decreased frontal and parietal activation during some working memory tasks (Dumas and Newhouse, 2015). Weakened prefrontal and frontal connections may suggest decreased activation of the cortico-limbic circuit, which is related to symptoms such anhedonia or blunted affect (Fingelkurts and Fingelkurts, 2006). Some studies found that local information flow in the frontal-parietal-occipital network was related to the level of sedation (Rathee et al., 2016). For most stroke patients, the main symptoms of depression are decreased interest and retardation, which may cause the performance in the frontal-parietal-occipital network to become similar to that with sedation.

Post-stroke depressed patients exhibit lower clustering coefficients and more diffuse distribution of core nodes. The hypothesis of nerve loop connectivity injury has been used to explain the incidence of depression in some studies. Specifically, the pathogenesis of depression has certain neuroanatomical mechanisms. Damage to certain brain-related areas disrupts the neural pathway of emotional regulation, resulting in depressive episodes (Greicius et al., 2007; Alexopoulos et al., 2008). Previous studies found abnormal connectivity of neural circuits in depressed subjects. Studies also found that antidepressant drugs can restore this connection, which identified the relationship between the incidence of depression and nerve connection disorders (Cai et al., 2013; Tadayonnejad et al., 2014; Gudayol-Ferre et al., 2015). We suggest that abnormal communication in emotion-related brain areas results in disconnection in PSD subjects, and this phenomenon is also related to the damaged “core node.”

Previous studies found dopamine-dependent changes in the functional connectivity between the basal ganglia and cerebral cortex (Williams et al., 2002). As depressive disorders were considered a syndrome of cortical-subcortical dysrhythmia (Fingelkurts and Fingelkurts, 2015), a basal ganglia lesion should disrupt the normal cortical-subcortical neural pathway, which regulates emotions. Our results support the conclusion that post-stroke depressed subjects demonstrated abnormal brain network connectivity and that network features determined based on EEG may be utilized as reliable biomarkers for the effective assessment of PSD in the future.

There are some limitations of the present study. Only 16 EEG channels were used in the present study, which limited the network nodes. We plan to collect 64-channel EEGs in the future to obtain a more precise network. Another limitation of the study was the different locations of hemispheric lesions in participants. This study contained both left and right hemispheric lesioned patients, which may confound the current results. As the left and right hemispheres have different roles in emotional processing, a depressed mood following different hemispheric lesions may result from different brain disconnections. In future studies, we plan to investigate differences in the brain network in post-stroke depressed subjects with left and right hemispheric lesions. There can be contamination of spurious connectivity caused by volume conduction using MI algorithm. We will try more methods such as phase lag index (Stam et al., 2010) and imaginary part of coherency (Nolte et al., 2004) to cope with this limitation in later studies.

## Ethics statement

This study was carried out in accordance with the recommendations of Tianjin Union Medical Center committee with written informed consent from all subjects. All subjects gave written informed consent in accordance with the Declaration of Helsinki. The protocol was approved by the Tianjin Union Medical Center committee.

## Author contributions

CS did the EEG analysis work and write the article. FY made all the figures and tables. CW worked for the depression diagnosis and severity assessment of poststroke patients. ZW did the work of statistical analyses of the data. YZ recruited all the subjects and made the selection for the study. DM verified the results of the article. JD designed the research plan and offered the electroencephalograph acquisition equipment.

### Conflict of interest statement

The authors declare that the research was conducted in the absence of any commercial or financial relationships that could be construed as a potential conflict of interest.
